# QOI5/394: A Stakeholder Matrix for Effective Health Web Design

**DOI:** 10.2196/jmir.1.suppl1.e98

**Published:** 1999-09-19

**Authors:** R Stevens

**Affiliations:** ^1^University of Salford, ManchesterUK

**Keywords:** Internet utilisation, Social Environment, Community Networks

## Abstract

**Introduction:**

Concern about health information quality on the Internet has focused on the 'demand side' - evaluation problems experienced by end-users. Less consideration has been given to the organisational circumstances in which health Web sites are created - the 'supply-side'. This project presents a case study of social and organisational factors affecting the development of a diabetes centre Web site.

**Methods:**

Action Research was used to develop a simple WWW site for Salford Diabetes Centre within Hope Hospital's 'Clinical Sciences Web'. A stakeholder matrix for evaluating IT products in diabetes care was used. Content was gathered from meetings, interviews, telephone calls, email messages and letters over a 2 year period. Style and content were kept simple so that: a) tight control over quality of information could be retained, and b) findings could be generalised to settings where technical expertise or network infrastructure may not be advanced. The Web designer was distanced from the diabetes centre to typify a 'consultancy' approach where external Web design services are 'bought in' because of expertise and technology provided, rather than specialist knowledge of, or integration within, the host organisation. A key hypothesis was that integration of the Web designer within the host organisation may be related to the quality of Web site produced.

**Results:**

1. Although Web site aims were limited, significant problems were experienced. These included: Gathering of content extremely difficult.
2. Web experience of health professionals began from low level.
3. Communication problems meant effective updating and editing of content seldom possible.
4. Web site perceived within organisation as single event rather than iterative process.
5. Reliance on content being provided by diabetes centre professionals led to other items not being covered.
6. Web site produced was rather dull and uninteresting - perhaps due to lack of a motivated project champion.
7. Feedback from staff difficult to gather. Perception of Web site as a 'good thing', but few positive contributions after initial stage.
8. Despite problems, diabetes centre remains positive about the Web site.
9. Drawing in other stakeholder groups was difficult, but the Gubbio matrix was a useful tool.

**Discussion:**

The evidence suggests the quality of a health Web site in meeting its aims may indeed be related to the web designer's degree of integration within the host organisation. However, it is possible that, if the aims are sufficiently limited, they can be met by a web designer who is not strongly integrated within the host organisation. For a 'corporate' site to be developed beyond a host organisation, a local stakeholder matrix may be useful.

